# Plant-Derived Chemicals: A Source of Inspiration for New Drugs

**DOI:** 10.3390/plants9111562

**Published:** 2020-11-13

**Authors:** Veronique Seidel

**Affiliations:** Natural Products Research Laboratory, Strathclyde Institute of Pharmacy and Biomedical Sciences, University of Strathclyde, Glasgow G4 0RE, UK; veronique.seidel@strath.ac.uk

**Keywords:** drugs, natural products, phytochemistry, plant-derived chemicals

## Abstract

Plants have a long history of use as traditional remedies to treat a range of diseases and the diverse chemicals that they produce have provided great inspiration for the design of new drugs to date. Many plants have yet to be investigated for the presence of biologically-active products. This Special Issue presents a collection of scientific studies which report on the medicinal potential of plants. It also highlights the importance of preserving ethnobotanical knowledge and plant biodiversity worldwide to sustain future drug discovery from plant sources.

Plants have a long history of use as traditional remedies to treat a variety of diseases, and produce a range of diverse chemical scaffolds that have specifically evolved to interact with biological targets. Many drugs based on plant-derived chemicals are currently employed in modern therapy. These include the anticancer drug paclitaxel from the yew tree (*Taxus*) species, the anticancer Vinca alkaloids from the Madagascar periwinkle (*Catharanthus roseus*), galanthamine from the common snowdrop (*Galanthus nivalis*) for the treatment of Alzheimer’s disease, and the antimalarial artemether derived from artemisinin isolated from the Chinese plant *Artemisia annua*. It has been estimated that only around 15% of plant species have been investigated phytochemically, and only 6% have been studied for their pharmacological potential. This means that large numbers of plant-derived chemicals with some pharmaceutical potential remain to be discovered [1,2,3]. 

This Special Issue on “Plant-Derived Chemicals: A Source of Inspiration for New Drugs” aims to add to the current knowledge on drug discovery from plants. In order to sustain future drug discovery efforts in this field, there are two key messages from the contributions presented in this Special Issue. The first one is that a variety of methodologies/approaches should be employed to discover new plant-derived medicines. The second is that it is vital that ethnobotanical knowledge and plant biodiversity are preserved worldwide for future discoveries of new biologically-active molecules from plant sources. 

## 1. Using Various Methodologies to Discover New Plant-Derived Medicines

Wali et al. used an in vivo study on rats to demonstrate the anti-oxidant, hepatoprotective, and anti-inflammatory activity of the flavonoid naringenin (mainly found in *Citrus* fruits). The aforementioned effects were observed upon administration of naringenin prior to treatment with the anticancer drug doxorubicin. The latter is known to increase the production of reactive oxygen and nitrogen species that can damage cells and lead to inflammation. The authors used a variety of biochemical tests to measure the levels of reactive oxygen species (ROS), lipid peroxidation products, anti-oxidant enzymes, and inflammatory mediators released in rats treated with naringenin and exposed to doxorubicin. Naringenin was found to decrease lipid peroxidation by diminishing membrane fluidity and lowering the production of ROS, increase anti-oxidant enzymes, and decrease the levels of inflammatory mediators. A further histological investigation on liver tissues confirmed the preventative effect of naringenin against doxorubicin-induced hepatotoxicity [4].

Di Ciaccio et al. evaluated extracts from *Peltophorum dubium* (Leguminosae) leaves collected in various locations within South America, for their antifungal activity against the mold *Aspergillus flavus*. This was performed using a combination of TLC bioautography, radial mycelium growth tests, and staining with Evans blue dye to record any changes in the morphology of *Aspergillus* hyphae. The authors concluded that *P. dubium* extracts altered the fungal cell wall and membrane. The presence of flavonoids, including kaempferol, naringenin, chrysin, daidzein and apigenin, was detected in the active extracts [5].

Shoaib et al. explored the neuroprotective properties of *Aconitum napellus* (Ranunculaceae) tubers using an in vivo model of streptozotocin-induced diabetic neuropathy. A series of tests were performed to measure body mass, blood sugar levels, oral glucose tolerance, hyperalgesia, cold allodynia, motor co-ordination, locomotor activity, and other relevant parameters. Histomorphology was used to assess the activity of *A. napellus* on the sciatic nerve. An in vitro assay was conducted on the human neuroblastoma cell line SHSY-5Y. A significant improvement in the myelination and degenerative changes of the nerve fibers along with behavioral changes were observed upon administration of *A. napellus,* suggesting that this plant contained some active chemicals worthy of further investigation [6].

Yarmolinski et al. studied the wound healing and anti-inflammatory properties of *Phlomis viscosa* (Lamiaceae) extracts using human dermal fibroblasts (in vitro scratch assay) and an in vivo wound healing assay in mice. A combination of three chemicals - diosmin, 1-octen-3-ol, and himachala-2,4-diene – isolated from this plant enhanced wound healing significantly both in vitro and in vivo. In addition, *P. viscosa* extracts and some of the isolated chemicals significantly decreased the secretion of pro-inflammatory cytokines by human dermal fibroblasts and inhibited the growth of several wound-associated microorganisms [7].

Qasaymeh et al. used an in silico target-based approach (molecular docking) to predict the binding affinity of several chemicals from *Pelargonium sidoides* and *P. reniforme* (Geraniaceae) on *M. tuberculosis* protein kinase G. The latter has been described as a good target for the discovery of new antitubercular drugs. The flavonoids orientin, populnin, and vitexin showed a high affinity for the target enzyme. Other chemicals with high efficiency indices included coumaric acid, coumaraldehyde, *p*-hydroxyphenyl acetic acid, p-hydroxybenzyl alcohol, methyl gallate, and the flavonoid myricetin [8].

## 2. Preserving Ethnobotanical Knowledge and Plant Biodiversity Worldwide

Ensuring that ethnobotanical knowledge is not lost, both in developing countries where plants continue to be widely used as traditional medicines and in the Western World, is important for the discovery of bioactive compounds from plants [9,10]. It is also vital that bioprospecting for plant-derived drugs is linked with sustained efforts to preserve plant biodiversity, which is currently in decline due to overexploitation of natural resources and global climate change [11].

The contribution from Blanco-Salas et al. was supported by a governmental initiative to valorize Spanish ethnobotanical knowledge and plant biodiversity. The authors reviewed the medicinal uses of *M. annua, M. ambigua, M. perennis*, and *M. tomentosa* (Euphorbiaceae) and their bioactive phytochemicals. The antidiabetic and antihypertensive properties of *Mercurialis* spp. were attributed to the flavonoids rutin and narcissin, respectively. Its anti-inflammatory activity was attributed to scopoletin, kaempferol, squalene, and cycloartenol [12].

## 3. Conclusions

Plant extracts and constituents continue to display interesting biological properties that provide inspiration for the development of new medicines. The discovery of plant-derived chemicals potentially useful for a range of therapeutic applications requires the use of a combination of approaches that involve ethnobotany, phytochemistry, medicinal chemistry, and pharmacology. Future discoveries in this field rely on the sustainable bioprospecting of plants.
